# 

**Published:** 1906-01

**Authors:** 


JANUARY, 1906.

### THE AMERICAN JOURNAL OF DENTAL SCIENCE.

EDITED BY
WM. GIRD BEECROFT, D. D. S., Madison, Wis.
B. H. TEAGUE, D. D. S., Aiken, S. C.

VOLUME 37. THIRD SERIES. NUMBER 1.

Published Monthly by
THE DENTAL PUBLISHING COMPANY,
Madison, Wisconsin.

The American Journal of Dental Science was founded in 1839 and is the oldest Dental Magazine in the world.

DENTAL HINTS,

The official organ of the Dental Societies of the South Atlantic States, and of the Chapin Harris Dental Society, has been merged with the Journal.

